# Obituary

**Published:** 1879-02

**Authors:** 


### Obituary.

ABSTRACT OF A MEMORIAL ADDRESS by Dr. Frederick Andros, delivered before the Northern Iowa Medical Association, Dec. 4, 1878, on the decease of the late DR. JOHN LINTON.

Dr. Linton was born in Breckenridge county, Kentucky, October 5th, 1811. When near his majority he apprenticed himself to a tanner and currier. After perfecting himself in his trade, he left the home of his nativity to seek his fortune in the wilds of the west. In the spring of 1837, he arrived at Prairie du Chien. Here he was employed by the Indian department, filling an important position, until 1842. He then returned to his native State and commenced the study of medicine under the tuition of Dr. Pollard, one of the prominent physicians of Kentucky. In the fall and winter of 1844 he attended medical lectures at the St. Louis University, where he was graduated. In the spring of 1846 he came to Garnavillo, in Clayton county, where he commenced the practice of his profession, and where he remained until his death.

His private life may be thrown freely open to the world, and challenge its closest scrutiny with a consciousness on the part of the critic that there is no blot to be concealed and no glaring fault which a love of truth bids him deny. Here was a man in whose professional or public life there can be found no instance of a mean or equivocating action, none of a departure from the self-imposed restraints of a lofty and refined sense of honor. He trod for thirty-two years the difficult and devious paths to professional preferment and success, and yet kept his robes unsullied by the mire that so often polutes those ways. Though lacking the advantages of a classical education, his life was an instance in which trne greatness was achieved without the aid of those brilliant qualities whose rare assemblage the world calls genius, but he possessed what is far better, a sound judgment and a resolute purpose to pursue the right and gather wisdom from experience. He had none of that presumptuous confidence in himself which so often misleads its possessor, and which, though dangerous, is still a commanding quality, yet he knew how to inspire the people with a just confidence in the soundness of his judgment and the integrity of his purpose, so as to be looked up to as a man in whom there was safety. But few men have lived among us whose lives have been as long and as active as his, who have secured more lasting friends (and none, I am sure, ever left fewer enemies). Nor was he one of those cold and impassive characters who shed their light without heat; its kindly influence, with a genial and friendly warmth was felt within whatever circle he might move. That man who, dying, can be said to have passed his days without a stain upon his reputation, has justly earned the honors due to a well spent life. Although he has passed from us forever, a fragrance still lingers around his memory, exhaled from the clustering virtues which beautified his character while living.

A sudden cold and over-exertion superinduced the disease which skill failed to alleviate, and on the 27th day of June last, his noble spirit winged its way to the country which we know not of. His religion was embraced in living up to the Golden Rule. The doctor was a firm and sincere believer in an overruling Providence, a Supreme Ruler of the universe, a God of love and mercy. Here was an example of the great truth that life, like the sword of Damocles, is suspended by a hair. May our lives be so ordered that when the great change shall come, our last hours like his may be peaceful and without doubt or fear when crossing the threshold of the unknown future.

---

THE FOLLOWING PREAMBLE and resolutions referring to the late A. W. LUECK, M.D., were passed at the last meeting of the Rock River Medical Society, at Fond du Lac, Wis., Jan. 8, 1879:

WHEREAS, We have learned with sincere sorrow, that death has removed from our midst our late esteemed friend and member, Dr. A. W. Lueck, of Maysville, Wis. Therefore

*Resolved*, That while we bow submissively to Him who doeth all things well, we yet sincerely deplore the early death of him whom we had learned to appreciate and love for his pure and noble character, and his unselfish devotion to the cause of science and suffering humanity.

*Resolved*, That his professional enthusiasm and success have afforded us unmistakable evidence of his fitness for the profession of his choice, and that his example is worthy of personal emulation by us all.

*Resolved*, That we shall ever hold him in remembrance for his genial social qualities, his unfeigned modesty and nobility of soul, and his professional integrity.

*Resolved*, That our earnest sympathy be extended to his sorrowing family, upon whom this severe affliction has fallen ; may they feel the tender care of the hand that has wounded but to heal, and be cheered by the thought that the life that has gone out so early in darkness, has even now entered upon an endless existence of joy.

*Resolved*, That the secretary be instructed to forward a copy of these resolutions to the friends of the deceased, and to the local papers, and the CHICAGO MEDICAL JOURNAL AND EXAMINER and to the *Medical and Surgical Reporter*.

N. SENN,<br>

H. P. WENZEL, } *Committee.*<br>

E. J. SMITH,

HENRY P. WENZEL, M.D.,<br>

HURESA, WIS., Jan. 9, 1879. Sec'y *Pro tem.*

---

THE METRIC SYSTEM IN ENGLAND.—The *British Medical Journal*, which is well known as the official journal of the British Medical Association, announces in its issue of Dec. 21, 1878, that it has applied to the American Metric Bureau for an explanation of the metric system, and prints two columns full of information derived from that source. We commend the enterprise of our cotemporary, but cannot repress the suspicion that he travelled a great distance in order to obtain his knowledge.

Said Dr. John Bull, of Great Britain—<br>

" With the French Metric System h'I'm smitten ;<br>

To the Yankees h'I'll go<br>

For to find h'out, y' know,<br>

'Ow the same by the Frenchmen h'is written!"

